# Promising Green Technology in Obtaining Functional Plant Preparations: Combined Enzyme-Assisted Supercritical Fluid Extraction of Flavonoids Isolation from *Medicago Sativa* Leaves

**DOI:** 10.3390/ma14112724

**Published:** 2021-05-21

**Authors:** Aneta Krakowska-Sieprawska, Katarzyna Rafińska, Justyna Walczak-Skierska, Anna Kiełbasa, Bogusław Buszewski

**Affiliations:** 1 Department of Environmental Chemistry and Bioanalytics, Faculty of Chemistry, Nicolaus Copernicus University, Gagarina 7 St., PL-87100 Torun, Poland; akra@doktorant.umk.pl (A.K.-S.); katraf@umk.pl (K.R.); kielbasam@umk.pl (A.K.); 2 Interdisciplinary Centre of Modern Technologies, Nicolaus Copernicus University, Wilenska 4 St., PL-87100 Torun, Poland; walczak-justyna@wp.pl

**Keywords:** enzymatic hydrolysis, EA-SFE, flavonoids, antioxidant, HPLC-MS/MS

## Abstract

To elaborate a complete extraction protocol for the enhanced release of biologically active compounds from plant cells, this study aimed to optimize together the parameters of the supercritical fluid extraction (SFE) process (temperature, pressure, and percentage of cosolvent) and enzymatic treatment of plant material (pH, enzyme concentration, time, and temperature) by response surface methodology (RSM). *Medicago sativa* L. was selected as a plant material due to its richness in phenolics and flavonoids. HPLC-MS/MS analysis allowed evaluating the content of individual bioactive compounds in obtained extracts. The total content of polyphenolic compounds in the extract obtained after two-step optimization was much higher (546 ± 21 µg/g) than in the extract obtained from non-hydrolyzed material (275 ± 23 µg/g) and in the extract obtained by maceration (162 ± 20 µg/g). Furthermore, it was evidenced that extract with the highest content of polyphenolic compounds can support the cellular antioxidant system both as a free radical scavenger and by stimulating the antioxidant enzyme system.

## 1. Introduction

Most biological active compounds are not directly involved in plant growth and development but play an essential role in plants’ interactions with the environment. The synthesis of these compounds is characteristic of higher plants and occurs only in specific plant tissues or organs. Due to the remarkable biological activity, the importance of secondary metabolites has increased [1,2,3]. The compounds like phenolics or saponins are not only the essential ingredients of food but are also highly desirable compounds in the pharmaceutical and cosmetics industries. They are commonly used as drugs, food additives, fine chemicals, or more recently in nutraceuticals [1,2].

The most common and most often studied polyphenols are flavonoid dye compounds that give flowers, fruits, and leaves color. The effect of the antioxidant properties of flavonoids is their pharmacological activity. These compounds show anti-inflammatory, anti-allergic, anticoagulant, anti-ulcer, diuretic, spasmolytic and anti-cancer effects [4,5].

Flavonoids and saponins are well-characterized classes of secondary metabolite classes produced by *Medicago sativa* L., commonly known as alfalfa, a perennial plant from the Fabaceae family. Cultivated since antiquity, it used both in cooking and in phytotherapy. Over the years, perceived only as a fodder plant, today it returns to favor due to the nutrients and bioactive substances it contains that can positively affect health. Due to its nutritional properties and rich vitamin and mineral composition, alfalfa can be considered a natural multivitamin. The above-ground parts of the plant are rich, e.g., in beta carotene, vitamins C, E, those from group B, and minerals, such as potassium, iron, calcium, magnesium, and silicon. In addition, due to the high content of chlorophyll, alfalfa also has a detoxifying effect and prevents cancers of the digestive system. Triterpene saponins in the root of this plant lower cholesterol without affecting the level of its good fraction (HDL), protect against arteriosclerosis and stimulate the regression of atherosclerosis. Furthermore, this species was approved by European Food Safety Authority (EFSA) as a safe dietary supplement [6,7,8,9]. Moreover, *M. sativa* is the most widely cultivated legume globally, and it is an essentially unlimited source of biologically active compounds for many branches of industry.

Secondary metabolites occur in small quantities in the plants, and there are more valuable than primary metabolites. However, because the concentration of biologically active substances in plant raw materials is low, many studies have been carried out to develop more effective and selective methods of their extraction [10,11,12,13,14]. Special attention was put on developing a chemical process that uses environmentally friendly solvents [15]. Increasing demanding environmental rules impose the need for new or improved extraction processes to avoid using toxic organic solvents [16,17,18].

In recent years, supercritical fluid extraction (SFE) has become an alternative to conventional solvent extraction methods to isolate valuable organic compounds. Supercritical extraction is much more technologically advanced than traditional extraction processes. The temperature and oxidation associated with the preparation processes are harmful to vitamins, enzymes, and many other active substances [19]. Considering the environmental friendliness of carbon dioxide, the supercritical extraction technique is a potential choice for isolating these valuable components [20]. Moreover, adding ethanol to scCO_2_ allows the extraction of compounds with a broader polarity range [8,15,16,17,21]. As a result of supercritical fluid extraction, plant extracts with the highest purity level are obtained, which are microbiologically safe and retain natural active ingredients [22].

A key role in isolating bioactive substances with a high concentration of antioxidant compounds plays the appropriate selection of extraction conditions. However, the extraction process of plant secondary metabolites as part of phytochemical or biological investigations presents specific challenges. Operational conditions have a crucial impact on the selectivity of the extraction process. The selection of appropriate conditions for the extraction process leads to obtaining products with the highest content of biologically active compounds and the lowest interfering substances [23]. The different statistical models are used to reduce the number of experiments and identify the interactions among experimental variables [24]. Optimization of extraction conditions can be carried out by using RSM when several variables impact the single response or multiple responses of interest [25]. The use of statistical planning is a key strategy to evaluate the applied parameters like temperature, pressure, type of solvent or cosolvent. It has been successfully used to promote the extraction of phytochemicals from plant materials, thus improving the biological activity of the extracts obtained [8,24,26].

Recently, using enzymes to degrade the plant cell wall has attracted much interest. Hydrolyzed cell wall facilitates solvent flow through the cells and the release of biologically active compounds. The application of enzymatic hydrolysis before extraction can improve extraction efficiency due to better mass transfer, reduced particle size, increased contact area, and improvement of solvent distribution [10,11]. Furthermore, the enzyme digestion of raw material reduces solvent consumption and extraction time [12].

Numerous enzyme-assisted extraction protocols are known in the literature. Cellulase is used to extract polyphenolic compounds from seeds and skins of citrus fruits [27]. Likewise, cellulase under the trade name Celluclast has been shown to be effective in extracting antioxidant phenolic compounds from grape pomace [28]. Cellulase and beta-glucosidase used separately significantly increase the extraction of polyphenolic compounds from guava leaves [29]. On the other hand, pumpkin tissue digested with cellulase is an excellent source of lycopene [30]. Dal Magro et al. [31] demonstrated the synergistic effect of the two enzymes Pectinex Ultra Clear and Lallzyme beta in the extraction of grape juice from *Vitis labrusca* L. The mixture used improved the yield of grape juice and led to increased content of polyphenolic compounds. However, plant cell walls are a highly organized structure that comprises many different polymers like cellulose, pectins, or hemicellulose. Therefore, applying mixtures of enzymes specific to various polymers is usually a more effective approach.

The main disadvantage of this method is the difficulty in selecting the appropriate enzymatic reaction conditions. Each of the enzymes has a specific range of pH, temperature, and other factors to function properly. These parameters impacting enzyme-assisted release of bioactive compounds need to be optimized for each specific extraction process [15,32,33]. Moreover, most of the enzyme-assisted extraction protocols developed are based on using pure enzyme preparations and mixtures, which have limited industrial applications due to their high cost. For our research, we used a multi-enzyme preparation designed to improve the digestibility of feed for pigs Kemzyme^®^, containing xylanase, beta-glucanase, cellulase, amylase, and protease. Our previous research confirmed that this preparation improves the efficiency of extraction of polyphenolic compounds from plant material [8,34]. In addition, the great advantage is that this preparation comes at a relatively low price (approximately EUR 1 per kg), which facilitates large-scale transfer.

This study aimed to evolve a new extraction protocol based on optimizing two coupled processes, an enzymatic treatment followed by extraction using clean technology viz supercritical fluid extraction. The mathematical and statistical method was used to selecting appropriate conditions for the extraction process and enzymatic hydrolysis. The obtained extract was tested for antioxidant properties and cytotoxicity. As mentioned before, all experiments and analyses were performed on *M. sativa* L. leaves. However, the developed methodology can be successfully implemented for any plant material containing pectin-cellulose cell walls. Results obtained from implementing this method based on green chemistry principles may play a key role in obtaining valuable products of high biological activity and potential application in the food, pharmaceutical, and cosmetic industry.

## 2. Materials and Methods

### 2.1. Chemicals and Reagents

The following chemicals and compounds were purchased from Sigma-Aldrich (Steinheim, Germany): polyphenolic standards (analytical grade; purity ≥ 99%), Folin–Ciocâlteu reagent, 2,2-diphenyl-1-picrylhydrazyl (DPPH), gallic acid, aluminum chloride, sodium carbonate and acetonitrile, formic acid, ethanol and methanol (all LC–MS grade). CO_2_ was 99.99% purity. Feed enzyme formulation (Kemzyme) was purchased from Kemin (Des Moines, IA, USA). Ultrapure water used to prepare blanks and standards solution was purified using a Milli-Q RG apparatus (Millipore InterTech, Bedford, MA, USA) in our laboratory. All other chemicals and reagents were of analytical reagent grade.

### 2.2. Plant Material

Plant material was produced in the Nicolaus Copernicus University, Torun, Poland, under the following conditions: (16 h day/8 h night; 21 °C). As shown by our previous research [7], the highest level of flavonoids is present in alfalfa leaves; therefore, extractions were performed with this part of *Medicago sativa* L. Alfalfa leaves were collected four weeks after sowing at the end of November in 2018 and dried in an oven (50 °C for 24 h). These materials were milled to a fine powder by laboratory mill (the average particle diameter was less than 1 mm) and were kept in the darkness until further use.

### 2.3. Extraction Procedure

Supercritical scCO_2_ extraction was performed by a laboratory-scale extractor (MV-10 ASFE Systems) supplied by Waters Corporation (Milford, MA, USA). To control the process, ChromScope^TM^ software was used. The sample (500 mg) was placed in extraction vessels (5 mL) and completed with glass beads.

To optimize SFE condition, the Box–Behnken design (BBD) (Design-Expert v.11 Trial, Stat-Ease, Minneapolis, MN, USA) was performed in the temperature range 50–70 °C and pressure from 100 to 300 bar, as well as with 10–20% percentage of added cosolvent (96% ethanol), as independent factors. Twelve runs and three replicates at a center point were executed to estimate these key factors. Each factor was examined at three levels (−1, 0, 1) in a randomized order, as presented in Table S1 (Supplementary materials). Total flavonoids content as response variable was fitted by the following second-order polynomial equation, describing the relationship between responses and independent variables:

(1) $$Y=b_{0}+\sum b_{i}X_{i}+\sum b_{ii}X_{ii}^{2}+\sum b_{ij}X_{i}X_{j}$$

where *Y* denotes the response variable; *X_i_* and *X_j_* are the independent variables (temperature, pressure, and percentage of added cosolvent); *b*_0_, *b_i_*, *b_ii_*, and *b_ij_* are the regression coefficients for intercept, linear, quadratic, and interaction terms, respectively.

The duration of one run of the extraction process was 30 min static time and 10 min dynamic mode (continual flow). Obtained extracts were stored at −20 °C until analysis. For comparison, maceration in optimal extraction parameters was carried out. Briefly, the plant material (500 mg) was soaked in ethanol (20 mL) and mixed for 24 h at the optimal temperature (50 °C) in the dark.

### 2.4. Determination of Total Flavonoids Content (TFC)

Total flavonoids content was determined by the aluminum chloride colorimetric method based on the technique described by Rouphael et al. [35] with modifications. Briefly, 62 µL of extract were mixed with the same volume of 2% AlCl_3_ in ethanol and next diluted with ethanol to 250 µL. After 40 min incubation at ambient temperature, absorbance was read at 415 nm in Varioskan^TM^ LUX multimode microplate reader (Thermo Fisher Scientific, Waltham, MA, USA) against the prepared blank. Measurements were performed based on a standard curve of rutin. The total flavonoid was expressed in mg of rutin equivalents per g of dry material (mg RE/g DW).

### 2.5. The Selection of Conditions for Enzymatic Hydrolysis

The procedure of enzymatic hydrolysis using kemzyme was described in our previous work [8]. In brief, 0.5 g of ground *M. sativa* L. leaves were diluted at the appropriate pH of 1.5 mL phosphate buffer (0.02 M), blended with the relevant concentration of kemzyme (2.9%), and incubated at the required temperature for a specific time, as presented in Table S2 (Supplementary materials). Afterward, the kemzyme was inactivated at 90 °C for 5 min and degassed in an ultrasonic bath (for 15 min). After this point, the enzyme-treated leaves were used for the SFE in optimized conditions. The specification of the enzyme is shown in Table S3 (Supplementary materials).

For optimization of crucial factors having a significant influence on enzymatic hydrolysis, i.e., pH, enzyme concentration, temperature, and reaction time, BBD was also applied. Altogether, 24 runs and three replicates at center points were performed. The four independent variable levels with coded values as a minimum (−1), center (0), and maximum (1) and physical values are shown in Table S2. For the description relationship among responses and independent variables, Equation (1) (Section 2.3) was applied. Optimal conditions for enzymatic hydrolysis were determined towards total flavonoid content as a variable response. Analysis of variance (ANOVA) with the significance level of 0.05 was performed as a statistic test for all obtained data.

### 2.6. HPLC-ESI-MS/MS Analysis of Polyphenolic Compounds

A Shimadzu LC–MS 8050 (Tokyo, Japan) was used to identify the phenolic compounds of the *Medicago sativa* extracts. The samples were separated using the Kinetex F5 column (100 × 2.1 mm, 2.6 µm, Phenomenex, Torrance, CA, USA). A 0.4 mL/min flow rate was used with a 10 min elution gradient, composed of 0.1% formic acid in water (A) and acetonitrile (B). The gradient was 0–7 min, 0–80% B at 7–8 min, 80–80% B at 8–10 min, 80–0% B. The injection volume for the sample was 10 µL. Lab Solution 5.8 software was used for instrument control, data acquisition, and processing. MS/MS analysis was performed in positive and negative ionization mode on a triple quadrupole in the m/z of 100 to 1000 (Shimadzu) equipped with an electrospray ionization (ESI) source. The optimal parameters of ESI-MS were as follows: detector voltage—1.2 kV, DL temperature—230 °C, heat block temperature—400 °C, nebulizing gas flow—3 L/min, heating gas flow. The purity of gas was 99.99%. The quantitative analysis was acquired using flavonoids and phenolic acids solutions at eight concentrations from 0.00005 to 10 μg/mL. All polyphenol compounds were monitored in the scheduled multiple reaction monitoring (MRM) mode. A list of all MRM transitions, collision energy, Q1, Q3, and dwell time for investigated phenolic compounds is shown in Table S4 (Supplementary materials). All quantitative analysis was described in our previous article [8]. The single MRM chromatograms and full chromatograms for the investigated phenolic compounds are shown in Figure S1.

### 2.7. Antioxidant Activity of the Extracts

#### 2.7.1. DPPH Method

By referring to our earlier paper [22], the free radical scavenging activity using the DPPH reagent was performed. The results were given as a Trolox equivalent (mg TEAC/g DW).

#### 2.7.2. AgNP Method

The antioxidant capacity of the obtained extracts was determined using the silver nanoparticle-based (AgNP) method described by Szydłowska-Czerniak et al. [36] with modifications. In brief, 10 µL of extract, 50 µL of ammonium buffer (pH 8.4), and 50 µL of 10 mM silver nitrate were mixed into a 96-well microplate and was made up with redistilled water to 250 µL. Then, the solutions were vigorously mixed and incubated in the dark for 30 min. The absorbance of these mixtures was measured at 405 nm, using a Varioskan^TM^ LUX multimode microplate reader (Thermo Fisher Scientific, Waltham, MA, USA) against a reagent blank (50 µL ammonium buffer and 50 µL of 10 mM silver nitrate made up with redistilled water to 250 µL).

Our previous work showed [12] that apigenin is the predominant flavonoid in *M. sativa* L. leaves; therefore, this compound was used as the reference standard. Measurements were calibrated to a standard curve with Equation 0.2913x + 0.020 (R^2^ = 0.9995) and range 0.09–3 μmol/mL. The results were expressed as μmol of apigenin equivalent (AP) per gram of dry material. The formation of silver AgNPs was confirmed by transmission electron microscopy (TEM) and energy-dispersive X-ray (EDX) analysis.

#### 2.7.3. Impact of EA-SFE *M. Sativa* L. Extract on Antioxidant Enzyme Activity GSH-Px

L929 normal mouse fibroblast cells used in the experiment were obtained from American Type Culture Collection and cultured in a humidified atmosphere of 5% CO_2_ at 37 °C. This cell line was routinely maintained in Dulbecco’s modified Eagle’s medium supplemented with 10% fetal bovine serum, 100 U/mL penicillin, and 100 μg/mL streptomycin, passaged by 0.25% trypsin/EDTA every 3–4 days and used in passage 8.

To create oxidative stress in cells, H_2_O_2_ was added. Damage of cells was checked by MTT assay. L929 cells (1 × 10^5^ cells/mL) were exposed to H_2_O_2_ at concentration 0.1–0.6 mM for 4 h. At the end of the incubation time, cells were incubated for 4 h with 0.5 mg/mL of MTT. After this, the medium was removed, and DMSO was added. Cells were gently shaken, and absorbance was measured at 560 nm using Varioscan^TM^ LUX Multimode Microplate Reader. For further experiments, the concentration 0.3 mM H_2_O_2_ was chosen as corresponding to about 70% cell viability measured by the MTT assay.

To examine the protective effects against H_2_O_2_–induced oxidative stress, cultured L929 cells were pretreated with different concentrations of *M. sativa* L. extract obtained by extraction in optimized conditions (0–0.5 mg/mL). After 24 h, cells were exposed to 0.3 mM H_2_O_2_ for 4 h and then harvested with trypsin, washed by PBS, and homogenized. The activity of GSH-Px was measured spectrophotometrically using kit ab102530 glutathione peroxidase assay kit (Abcam). The enzyme-specific activity was calculated in units per mg of protein. Direct Detect^®^ Infrared spectrometer measured the protein content according to the calibration curve to bovine serum albumin.

## 3. Results and Discussion

### 3.1. The Selection of Supercritical Fluid Extraction Conditions

The extraction process under supercritical conditions is much faster compared to the classical liquid extraction processes. This technique allows the control of the process parameters and ensures high selectivity and separation of extraction products. The extraction rate depends on the density of the supercritical fluid, which is pressure and temperature-dependent. The scCO_2_ density decreases with increasing temperature. However, at higher temperatures but keeping the same density, the extraction process is faster [37,38,39]. With increasing temperature, both the thermal conductivity and diffusivity in supercritical carbon dioxide increase [40].

Carbon dioxide is a completely non-flammable and nontoxic gas and is readily used to extract valuable plant compounds. It is easy to control its ability to dissolve various substances by changing the process parameters. This possibility is not offered by traditional solid–liquid extraction methods [15]. Carbon dioxide does not dissolve some compounds, such as phenols, alkaloids, or glycosides, due to their polar nature. Still, a small addition of organic solvents allows increasing the efficiency of the extraction process of polar compounds [37,39].

The RSM was applied to optimize the SFE conditions from *M. sativa* leaves for the highest bioactive compounds, especially flavonoids. The effect of extraction temperature (50, 60, and 70 °C), pressure (100, 200, and 300 bar), and cosolvent content (10, 15, and 20% ethanol) was investigated as independent variables to enhance the total flavonoid content (Table S1).

As can be seen, in different examined extraction conditions, total flavonoid content (TFC) in alfalfa leaves extract varied from 0.10 to 2.12 mg RE/g DW. In this research, the highest flavonoid content was obtained at 50 °C, under pressure 200 bar, with 20% ethanol as cosolvent. On the other hand, the lowest TFC value was obtained for the extract obtained at 60 °C, pressure 100 bar, and 10% addition of cosolvent. The calculated R^2^ value was 0.9940, indicating a very good fit between experimental and theoretical values. The model’s efficiency was also confirmed by a low coefficient of variation (CV) value of 6.48%. The influence of independent variables on TFC in alfalfa extracts was also described and predicted by the second-order polynomial equation (Table 1). The mathematical model applied for the response was statistically acceptable due to significant regression for the model (*p* < 0.05) and the lack of fit (*p* > 0.05) (Table 1).

**Figure 1:**
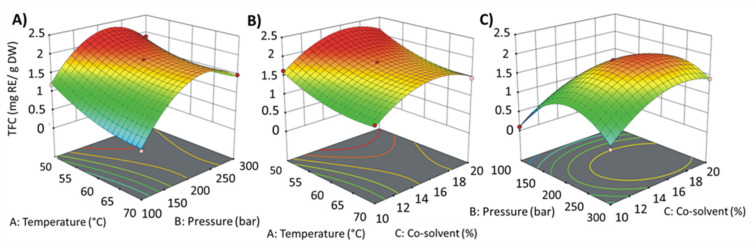
Response surface contour plots showing combined effects of extraction parameters on total flavonoid content (TFC). (**A**) temperature and pressure; (**B**) temperature and cosolvent; (**C**) pressure and cosolvent.

Figure 1 shows the influence of all three extraction parameters on the TFC in the obtained extracts. The addition of a cosolvent is considered one of the most influential parameters for the flavonoid extraction process. On top of it, it can be seen that the flavonoids content in the observed extracts increased with increasing temperature to about 60 °C. Further increase in temperature resulted in decreased TFC. In general, increased temperature allows greater penetration of scCO_2_ as it reduces the viscosity and surface tension of the water contained in the plant. Moreover, as the temperature increases, the diffusion of bioactive substances into CO_2_ is greatly facilitated, and the vapor pressure of the solute increases. However, an excessively sharp temperature rise may reduce the solvent density, reducing the solubility of the compounds under study [41].

The increase in pressure increased the content of flavonoids. The results observed during the present attempt are comparable with a study conducted by Goyeneche et al. [42] in extracting polyphenols from beetroot leaves. With increasing pressure, kinetics (rate) also increased due to the increase in scCO_2_ density.

After calculation by Design Expert 11 software, the optimal parameters of TFC extraction for the three independent variables (temperature, pressure, and% of cosolvent) were 50 °C, 216 bar and the cosolvent share of 19.4%, with a corresponding Y (TFC) of 2.28 mg RE/g DW (Table 2). To confirm the results, extraction was carried out under optimized conditions. The TFC was 2.15 mg RE/g DW, which exhibited that the model fitted the experimental data.

### 3.2. The Effect of Enzymatic Hydrolysis Conditions on the Total Flavonoid Content (TFC) in M. sativa Leaves Extracts

Some bioactive compounds are bound by chemical bonds in plant matrices, especially with plant cell walls, and are difficult to isolate through routine extraction using solvents. In our research, we proposed an enzymatic pretreatment of plant material to improve the extraction efficiency of bioactive compounds. The enzymes used break down the polysaccharide structure of the cell wall [43].

The enzymatic reactions are influenced by chemical and physical factors, such as enzyme concentration, temperature, hydrogen ion concentration (pH), activators, and inhibitors. Moreover, Klason lignin present on the surface of cellulose may significantly limit the area to be accessible for hydrolytic enzymes, reducing the catalytic efficiency of enzymes [44]. Hence, it is necessary to optimize key factors that significantly impact the quality of hydrolysis, i.e., pH, enzyme concentration, temperature, and reaction time [45].

One of the key factors to consider for selecting the optimal conditions for enzymatic hydrolysis is combining time and temperature. The influence of temperature on enzyme activity is not a simple relationship. Activity increases with increasing temperature, but only over the temperature range where the enzyme remains stable. When the critical temperature is exceeded, the enzymes are thermally denatured. As a result, of which their activity drops sharply. Temperature control is essential in the extraction of polyphenolic compounds, as these are compounds that decompose quickly at high temperatures [46,47]. The optimum temperature for most enzymes is in the range of 30–45 °C and irreversibly denature and lose their activity at temperatures higher than 60 °C. Operation at these temperatures causes both a gradual loss of enzymatic activity and the inactivation of proteins and other biologically active compounds. Moreover, most enzymes slowly denature even at optimal and below critical temperatures. It depends on the nature of the enzyme itself, pH, ionic strength, and other parameters [48,49].

The optimum pH, next to the optimal temperature, is the second most important parameter characterizing the activity of enzymes. The influence of pH on the activity of enzymes is related to the fact that enzymes as proteins have many ionizable amino acids, and active center amino acids can often play their role only in a certain ionization state. In the optimal pH, the velocity of the catalyzed reaction is maximal. However, below or above the optimum pH value, this velocity declines [48]. Furthermore, for many enzymes, the optimal pH is in the range of the isoelectric point of proteins. In this range, the proteins are highly insoluble, which means that the release of the bioactive compounds can be hindered [47,49].

To investigate the effect of enzymatic hydrolysis conditions, in this case, pH (4–6–8), the concentration of enzyme used (2–3–4%), incubation time (30–60–90 min) and incubation temperature (30–40–50 °C) on the TFC, the RSM methodology was used. The experimental results of the tested response using the BBD project are present in Table S2.

Table 1 summarizes the ANOVA (test F) and the value of p, which is used to check the significance of each factor and indicate the strength of interaction of each parameter. In this experiment, a model with a *p*-value less than 0.0001 was statistically significant. The “lack of fit” of this model was insignificant. The *p*-value was 0.0515, which suggests that the model was appropriate for this experiment. The determination coefficient (R^2^) and the adjusted coefficient of determination (Adj. R^2^) were 0.9983 and 0.9964, respectively, which indicates that the accuracy and overall availability of the polynomial model are appropriate. Moreover, these results showed that the second-order polynomial model represents an excellent approximation of experimental results.

The various flavonoids were obtained when different enzymatic hydrolysis parameters applied, ranging from 0.48–3.96 mg RE/g DW (Table S2). The maximum TFC content was observed for the central point, i.e., at pH = 6, for 3% enzyme concentration, for 60 min incubation at 40 °C. On the other hand, the increase in pH and time caused the lowest TFC value.

**Figure 2:**
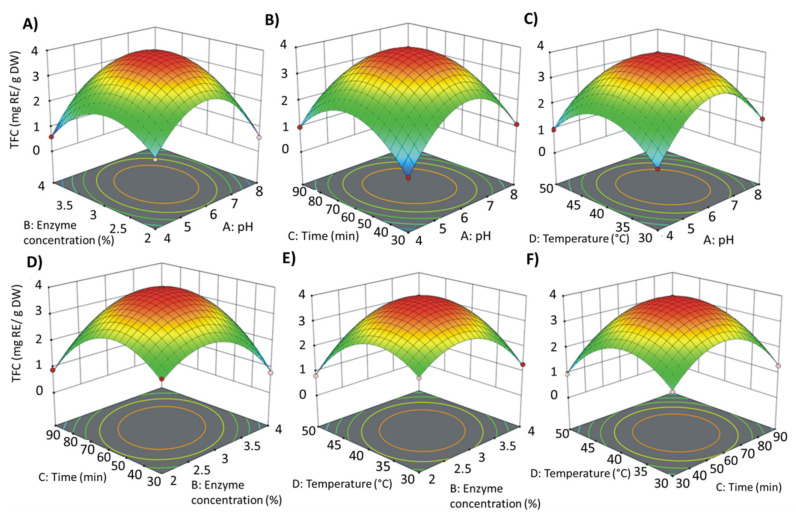
Response surface contour showing combined effects of enzymatic treatment conditions on flavonoid content (TFC). (**A**) enzyme concentration and pH; (**B**) time and pH; (**C**) temperature and pH; (**D**) time and enzyme concentration; (**E**) temperature and enzyme concentration; (**F**) temperature and time.

Based on the regression model analysis results obtained with the Design-Expert 11 software, three-dimensional graphs were drawn (Figure 2). It can be seen that temperature, time, and enzyme concentration were parameters that had a significant influence on the flavonoids content of the obtained extracts. Our results showed that pH was an insignificant parameter. However, this surprising result may be because a mixture of as many as five enzymes was optimized, each of which has its own optimum of activity. Therefore, the relationship between pH and flavonoids extraction may be nonlinear. In the case of optimization of the activity of one enzyme, the pH parameter should be significant, as has already been proven by many studies.

The optimal conditions for enzymatic hydrolysis are summarized in Table 2. The results observed during the present attempt are comparable with a previous study conducted by Mushtaq et al. [11]. They observed that for Kemzyme, the optimal temperature and incubation time were 35 °C and 60 min, and pH in range 4–6. For verification, extraction was carried out using enzymatic hydrolysis under optimal conditions. The predicted content of flavonoids should be 3.96 mg RE/g DW. The obtained extract this value was 3.89 mg RE/g DW, which confirms optimizations accuracy because the obtained result was in the confidence interval.

### 3.3. Chemical Analysis of Obtained Extracts by HPLC-ESI-MS/MS

One of the largest groups of secondary metabolites that carries various biological functions in plants is flavonoids. These substances function as a dye; they protect the plant from the harmful effects of UV radiation and natural insecticides and fungicides [50,51]. Due to hydroxyl groups, double bonds, and carbonyl groups in their structure, flavonoids possess strong antioxidant activity. The antioxidant activity of flavonoids is to capture free oxygen radicals and their reactive forms and reduce their production by inhibiting the activity of enzymes involved in ROSe production [8,51,52]. These compounds, a component of dietary supplements, are an integral part of cosmetic and functional food formulas. The main problem in obtaining them is their low level in plant material and the difficulty of extraction. The synthesis and storage of plant biologically active substances are multi-stage processes. They take place both in the intracellular membrane system of plant cells and in the area of cell walls [53]. Hence, some biologically active compounds are localized intracellularly. Some are bound by weak interactions, e.g., hydrophilic or hydrogen bonds with components of cell walls, e.g., pectins, cellulose and hemicellulose. As a result, it is difficult to wash out them using conventional extraction techniques and traditional solvents. The extraction processes carried out are characterized by low yields [54].

The *M. sativa* leaves were extracted by maceration, SFE, and EA-SFE after enzymatic hydrolysis in optimal conditions. The chemical profile of extract samples obtained by HPLC-ESI-MS/MS analysis is presented in Table 3.

**Figure 3:**
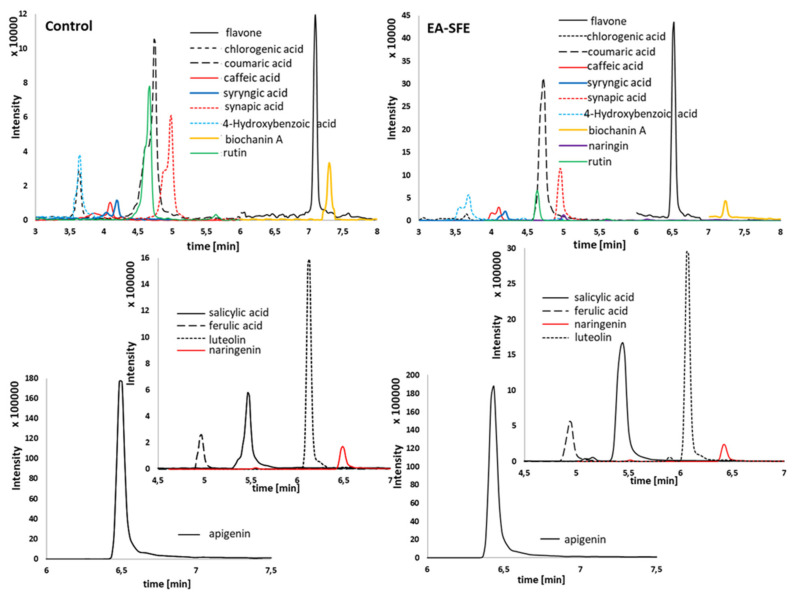
HPLC-MS/MS chromatograms of selected main compounds from control and EA-SFE extracts.

The EA-SFE was proven to be an excellent choice for the extraction of both phenolic acids and flavonoids. HPLC-MS/MS analysis showed that in extracts obtained from material previously subjected to enzymatic hydrolysis under optimal conditions, the obtained summary content of phenolic compounds was approximately 2-and 3.4-times higher than the extracts from the control and maceration, respectively. With apigenin and salicylic acid, ferulic acid was present in the highest concentration in the obtained extracts (Figure 3). In addition, a very high increase in concentration was observed for both phenolic acids, thanks to using enzymatic hydrolysis with optimal conditions before supercritical fluid extraction. Indeed, the higher liberation of phenolic compounds may be attributed to the compositional profile of enzyme preparation. This is confirmed by our previous research [8]. However, optimizing conditions of enzymatic hydrolysis was the main factor affecting this process.

### 3.4. Antioxidant Activity of the Extracts

**Figure 4:**
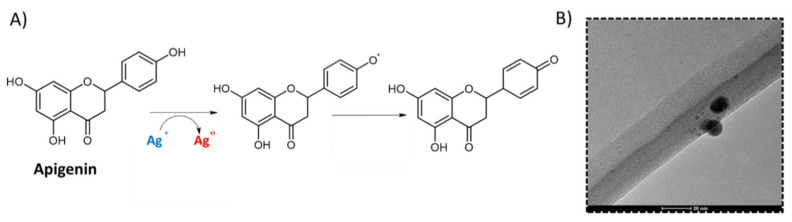
The possible chemical reaction for the AgNP method (**A**) and TEM micrographs of the formed silver nanoparticles (AgNPs) (**B**).

There are numerous methods and modifications for the estimation of antioxidant activity. In our research, antioxidant activity was determined by two spectrophotometric assays. The first is the commonly known method using the DPPH reagent, in which the extract reacts with DPPH radical solution in ethanol, which leads to reducing the DPPH. The decrease in DPPH concentration is measured at a characteristic wavelength of 517 nm [55,56]. The second method allows the determination of the antioxidant activity through the ability to reduce silver ions and forming silver nanoparticles (AgNPs). This method is based on the growth of silver nanoparticles through phenolic compounds characterized by high antioxidant action (Figure 4A). The AgNP method is not only a simple, fast, precise, and reliable method for determining antioxidant activity but also raises interest due to the ecological and “green” biosynthesis of silver nanoparticles [30,57].

To confirm the formation of AgNPs in our study, the TEM technique was used and showed that the formed AgNPs were spherical shaped (Figure 4B). In turn, EDX analysis clearly showed the presence of an elemental silver signal of the AgNPs. This confirmed the w % mass percentage of the analyzed element.

The results of antioxidant activity determined by the modified AgNP and DPPH method in *M. sativa* extracts obtained by different extraction techniques are shown in Table 3. Antioxidant activities of the studied extracts range between 0.85 and 1.71 µmol TEAC/g DW for the DPPH assay and 20.50–27.36 µmol AP/g DW for the AgNP method. Results indicate that extracts obtained by EA-SFE exhibit higher antioxidant potential than extracts from non-hydrolyzed material, in the case of both tested methods. Furthermore, the antioxidant capacities determined by DPPH and AgNPs methods were significantly correlated with the number of phenolic acids and flavonoids in obtained extracts. The extracts obtained by EA-SFE that particularly rich in phenolic acids were characterized by the highest antioxidant potentials. We can conclude that extracts obtained after enzymatic pretreatment will significantly inhibit ROS formation, whose excess causes oxidative stress [58]. This, in turn, confirms the effectiveness of the extraction method used, which should mainly be checked in terms of the quality of the obtained extracts, namely their ability to inhibit or regulate the oxidation process [11].

**Figure 5:**
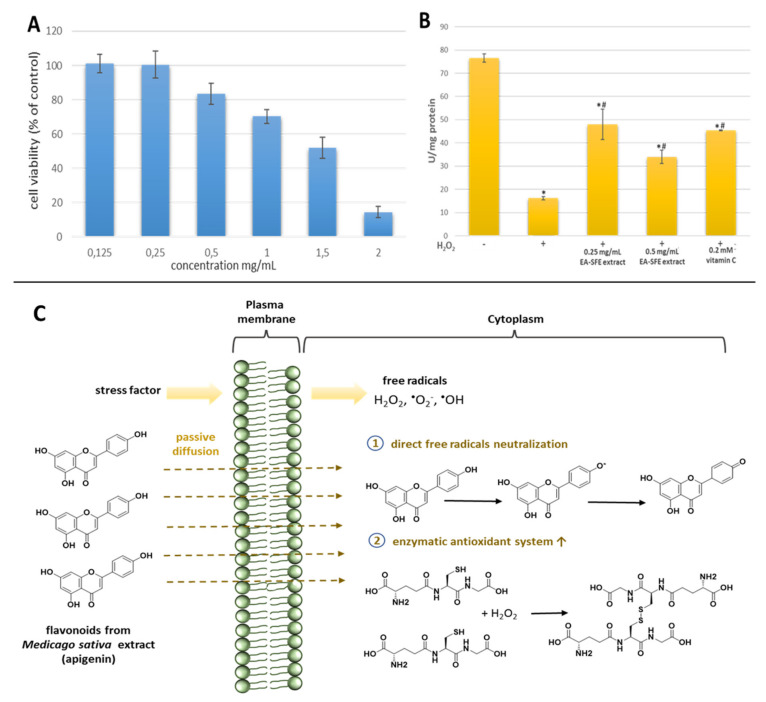
Characteristic of EA-SFE *M. sativa* L. extract cytotoxicity (**A**) and effect of EA-SFE *M. sativa* L. extract on GSH-PX activity H_2_O_2_-treated L929 cells. * *p* < 0.05, versus a standard control group. # *p* <0.05, versus the H_2_O_2_ control group (**B**) and mechanism of antioxidant activity of *M. sativa* L. flavonoids (**C**).

Finally, we checked the cytotoxicity and ability of EA-SFE extract to control oxidative stress in living cells (Figure 5A,B). A cytotoxicity test showed that in the range of concentration up to 0.5 mg/mL, the tested extract did not significantly reduce L929 cell viability (Figure 5A). The IC_50_ value was 1.36 ± 0.015 mg/mL. Generally, cytotoxicity of EA-SFE *M. sativa* extracts obtained after one-step optimization based on selecting extraction parameters for phenolic compounds showed slightly lower cytotoxicity due to a higher level of biologically active compounds in extract after two-step optimization [8]. The beneficial effect of the compounds depends on the dose. It is well-known that too high a concentration will have undesirable effects. In the second step, the impact of *M. sativa* extract on the enzyme antioxidant system in live cells was studied. As an example of an oxidative stress biomarker, glutathione peroxidase (GPx) that protects cells from oxidative damage was used. This family of enzymes converts reduced glutathione (GSH) to oxidized glutathione (GSSG) and during this process reduces lipid hydroperoxides to their corresponding alcohols or hydrogen peroxide to water. As shown in Figure 5B, incubation of L292 normal fibroblast cell line with H_2_O_2_ resulted in the lower activity of GSH-Px than the untreated group (*p* < 0.05). However, adding EA-SFE extract attenuated the drop of GSH-Px activity. The activity of 0.25 mg/mL of EA-SFE *M. sativa* was comparable with the activity of 0.2 mM vitamin C. Similar, studies on RAW 264.7 indicate that pure vitamin C significantly attenuates the changes in GSH-Px activity. Moreover, vitamin C increased the expression level of GSH-Px in H_2_O_2_ pretreated cells. Therefore, the impact of plant extracts on the level of expression of important from a clinical point of view enzymes should be clarified in be the future. However, we can conclude that EA-SFE extract from *M. sativa* can support the cellular antioxidant system both as a free radical scavenger and by stimulating antioxidant enzyme (Figure 5C).

## 4. Conclusions

Recent trends set by the so-called “green chemistry principles” in extraction methods focus primarily on finding solutions that reduce solvent consumption and improve the efficiency of the process. One of them is to use enzyme-assisted extraction. To define the impact of extraction parameters of the SFE process and conditions of the enzymatic hydrolysis on the isolation of bioactive compounds, optimizing both processes were performed. In the first step, the influence of temperature, pressure, and cosolvent content was explored. The optimal combination of these extraction parameters was defined as 50 °C, 216 bar and 19.4% of cosolvent. Then, the optimal conditions for enzymatic hydrolysis by kemzyme were defined: pH 6, the concentration of enzyme 2.96%, incubation time 58.92 min, and incubation temperature 38.96 °C. Chemical profiles of the tested extracts were determined by HPLC-MS/MS. The 23 compounds were identified and quantified. This analysis confirmed that the summary content of phenolic compounds in the extract obtained by EA-SFE (546 ± 21 µg/g) was approximately 2- and 3.4-times higher compared to the extracts from control (275 ± 23 µg/g) and maceration (162 ± 20 µg/g), respectively. This combination of enzymatic hydrolysis process with SFE led to producing extract with a higher concentration of total polyphenolic compounds and particularly richer in apigenin, salicylic acid, and ferulic acid. At the same time, we have shown that the extracts obtained by EA-SFE with the highest concentration of flavonoids can support the cellular antioxidant system, directly neutralizing free radicals or activating enzymatic antioxidative mechanisms, which indicates a great practical application in different branches of industry.

Our work offers an effective protocol for producing extracts from *M. sativa* L. leaves with the highest bioactivity, which can then be used in the food, pharmaceutical, and cosmetics industries. However, the developed methodology can be successfully implemented for any plant material. The exploitation of EA-SFE in the industry to extract bioactive compounds from plant material is economically efficient and represents an advance in modern technological processes.

The methodology used allowed to maximize the recovery of biologically active compounds from plant material. In addition, analysis of obtained results notes how a small pool of the total content of these compounds in plant material has been extracted so far. We still do **no**t really know how many phenolic compounds and flavonoids are present in plant tissues and what percentage of them we can isolate. It seems reasonable to undertake further research on improving the efficiency of extraction.

## Figures and Tables

**Table 1:** Analysis of variance (ANOVA) of the fitted second-order polynomial model for total flavonoids content (TFC).

Source	Sum of Squares	Degrees of Freedom	Mean of Square	*F*-Value	*p*-Value
Selection of SFE Conditions					
**TFC**					
Model	5.77	9	0.6416	92.68	<0.0001 (significant)
Residual	0.0346	5	0.0069		
Lack of fit	0.0328	3	0.0109	11.74	0.0795 (not significant)
Pure error	0.0019	2	0.0009		
Total	5.81	14			
* Y (TFC) = 1.85 − 0.29X_1_ + 0.40X_2_ + 0.26X_3_ + 0.10X_1_X_2_ − 0.03X_1_X_3_ + 0.15X_2_X_3_ + 0.19X_1_^2^ − 0.79X_2_^2^0.45X_3_^2^					
**Selection of Enzymatic Hydrolysis Conditions**					
**TFC**					
Model	26.13	14	1.87	515.06	<0.0001 (significant)
Residual	0.0435	12	0.0036		
Lack of fit	0.0430	10	0.0043	18.80	0.0515 (not significant)
Pure error	0.0005	2	0.0002		
Total	26.17	26			
** Y (TFC) = 3.94 − 0.013X_1_ − 0.040X_2_ − 0.06X_3_ − 0.23X_4_ + 0.33X_1_X_2_ − 0.23X_1_X_3_ − 0.22X_1_X_4_ + 0.51X_2_X_3_ + 0.45X_2_X_4_ + 0.19X_3_X_4_ − 1.78X_1_^2^ − 1.23X_2_^2^ − 1.38X_3_^2^ − 1.23X_4_^2^					

* X_1_: extraction temperature (°C); X_2_: extraction pressure (bar); X_3_: percentage of cosolvent (%); ** X_1_: pH; X_2_: enzyme concentration (%); X_3_: time (min); X_4_: temperature (°C); TFC: total flavonoids content; *p*-values less than 0.05 indicate model terms all significant.

**Table 2:** Optimal conditions of the SFE and enzymatic treatment for total flavonoids content (TFC) as response and comparing predicted and experimental values (mg RE/g DW).

Optimal Conditions of the SFE								
Response Variable	Temperature (°C)	Pressure (bar)	Cosolvent (%)	Predicted Value	Experimental Value	Confidence Interval		
	Desirability-1.0					−95%		95%
TFC (mg RE/g DW)	50	216	19.4	2.28	2.15	2.11		2.45
**Optimal Conditions of the Enzymatic Treatment**								
**Response Variable**	**pH**	**Enzyme Concentration (%)**	**Time** **(min)**	**Temperature (°C)**	**Predicted Value**	**Experimental Value**	**Confidence Interval**	
	**Desirability-1.0**						**−95%**	**95%**
TFC (mg RE/g DW)	6.00	2.96	58.92	38.96	3.96	3.89	3.88	4.03

**Table 3:** HPLC-MS/MS determination of polyphenolic compounds and antioxidant activity in the maceration, SFE and EA-SFE extracts of *M. sativa* leaves.

HPLC-MS/MS Determination					
Compound	t_R_ (min)	MRM	Concentration (µg/g)		
			Maceration	SFE (Control)	EA-SFE (Optimal Conditions)
Phenolic acids					
Gallic acid ^+^	2.138	169–124	ND ^a^	ND	ND
Salicylic acid ^+^	3.961	137–93	6.79 ± 0.62	26.50 ± 2.94	100.48 ± 2.53
p-Coumaric acid ^+^	3.450	163–119	0.83 ± 0.34	1.34 ± 0.33	3.78 ± 0.10
Chlorogenic acid ^+^	2.681	353–191	ND	0.32 ± 0.14	0.13 ± 0.04
Caffeic acid ^+^	3.036	179–135	0.59 ± 0.22	0.92 ± 0.05	2.44 ± 0.11
Syringic acid ^+^	3.118	197–95	3.34 ± 0.99	4.73 ± 1.12	14.74 ± 2.77
Ferulic acid ^+^	3.566	193–134	56.36 ± 9.43	126.93 ± 6.99	277.41 ± 7.87
Protocatechuic acid ^+^	2.482	153–108	11.72 ± 2.78	2.82 ± 0.12	5.73 ± 1.89
Sinapic acid ^+^	3.553	223–121	3.03 ± 1.65	7.88 ± 3.57	9.28 ± 0.39
4-Hydroxybenzoic acid ^+^	2.890	137–65	4.48 ± 1.07	6.17 ± 2.22	20.80 ± 2.76
⅀			87.14 ± 17.10	177.61 ± 17.48	434.78 ± 18.46
Flavonoids					
Flavone ^++^	5.452	223–121	0.01 ± 0.00	0.23 ± 0.02	0.16 ± 0.01
Fisetin ^+^	3.928	285–121	ND	ND	ND
Kaempferol ^+^	4.811	285–255	ND	ND	ND
Apigenin ^+^	4.730	269–117	63.09 ± 1.33	87.48 ± 4.32	95.53 ± 1.19
Luteolin ^+^	4.359	285–133	9.12 ± 0.48	5.15 ± 0.23	9.86 ± 1.09
Rutin ^+^	3.447	609–300	0.62 ± 0.12	0.92 ± 0.13	0.49 ± 0.17
Quercetin ^+^	4.382	301–227	0.02 ± 0.00	0.61 ± 0.04	1.02 ± 0.23
Naringin ^+^	3.528	579–271	ND	ND	0.15 ± 0.01
Naringenin ^+^	4.744	271–119	1.39 ± 0.70	2.09 ± 0.13	2.53 ± 0.10
Esculin ^+^	2.492	339–177	ND	0.01 ± 0.00	0.01 ± 0.00
Esculetin ^+^	3.053	177–89	0.41 ± 0.06	0.37 ± 0.11	1.10 ± 0.13
Biochanin A ^+^	5.666	283–211	0.06 ± 0.01	0.21 ± 0.04	0.21 ± 0.01
Catechin ^+^	3.012	289–123	ND	ND	ND
⅀			74.72 ± 2.70	97.07 ± 5.02	111.06 ± 2.94
⅀			161.86 ± 19.80	274.68 ± 22.50	545.84 ± 21.40
**Antioxidant activity**					
AgNP method (µmol AP/g DW)			22.91 ± 0.81	20.50 ± 0.81	27.36 ± 0.75
DPPH method (µmol TEAC/g DW)			0.85 ± 0.01	0.97 ± 0.02	1.71 ± 0.08

^a^ ND—not detected; ^+^—negative ionization mode; ^++^—positive ionization mode. All results were expressed as mean ± standard deviation (*n* = 3) in µg per gram of dry leaves; SFE—supercritical fluid extraction; EA-SFE—enzyme-assisted supercritical fluid extraction; AP- apigenin; TEAC—Trolox.

## Data Availability

Not applicable.

## Supplementary Materials

The following are available online at https://www.mdpi.com/article/10.3390/ma14112724/s1, Table S1: Experimental conditions using in Box–Behnken design with coded and real values of independent variables (temperature; pressure; cosolvent content) and experimentally obtained values of total flavonoids content (TFC); Table S2: Experimental conditions using in Box–Behnken design with coded and real values of independent variables (pH, enzyme concentration, time and temperature) and experimentally obtained values of total flavonoids content (TFC); Table S3: Preferential enzymes and major active unit of enzyme formulation used; Table S4: MRM transitions, collision energy, Q1, Q3 and dwell time for investigated phenolic compounds; Figure S1: The single MRM chromatograms and full chromatogram for investigated phenolic compounds
