# Supplementary material for: Promising Green Technology in Obtaining Functional Plant Preparations: Combined Enzyme-Assisted Supercritical Fluid Extraction of Flavonoids Isolation from Medicago Sativa Leaves
Source: Materials (Basel). 2021 May 21;14(11):2724. doi: 10.3390/ma14112724 (PMC8196795; doi:10.3390/ma14112724)
Supplement: Supplementary file 1 [file materials-14-02724-s001.zip › materials-1210383-supplementary.pdf]

Supplementary Materials

Table S1. Experimental conditions using in Box-Behnken design with coded and real values of independent variables (temperature; pressure; co-solvent content) and experimentally obtained values of total flavonoids content (TFC)

Run Order	Independent Variables (coded and real values)			Measured Response
	Temperature [°C]	Pressure [bar]	Co-solvent [%]	TFC (mg RE/g DW)
	(X_1)	(X_2)	(X_3)	
1	-1 (50)	-1 (100)	0 (15)	1.19
2	1 (70)	0 (200)	1 (20)	1.48
3	0 (60)	0 (200)	0 (15)	1.81
4	0 (60)	-1 (100)	-1 (10)	0.10
5	-1 (50)	1 (300)	0 (15)	1.88
6	-1 (50)	0 (200)	1 (20)	2.12
7	1 (70)	1 (300)	0 (15)	1.49
8	1 (70)	-1 (100)	0 (15)	0.40
9	0 (60)	0 (200)	0 (15)	1.86
10	-1 (50)	0 (200)	-1 (10)	1.63
11	0 (60)	-1 (100)	1 (20)	0.41
12	0 (60)	0 (200)	0 (15)	1.87
13	1 (70)	0 (200)	-1 (10)	1.11
14	0 (60)	1 (300)	-1 (10)	0.51
15	0 (60)	1 (300)	1 (20)	1.41

Table S2. Experimental conditions using in Box-Behnken design with coded and real values of independent variables (pH, enzyme concentration, time and temperature) and experimentally obtained values of total flavonoids content (TFC)

**Run Order**	**Independent Variables (coded and real values)**				**Measured Response**
	**pH**	**Enzyme Concentration [%]**	**Time [min]**	**Temperature [°C]**	**TFC [mg RE/g DW]**
	**(X_1)**	**(X_2)**	**(X_3)**	**(X_4)**	
1	0 (6)	0 (3)	0 (60)	0 (40)	3.95
2	-1 (4)	1 (4)	0 (60)	0 (40)	0.59
3	0 (6)	1 (4)	0 (60)	1 (50)	1.71
4	-1 (4)	0 (3)	0 (60)	-1 (30)	0.98
5	0 (6)	0 (3)	0 (60)	0 (40)	3.93
6	0 (6)	0 (3)	-1 (30)	-1 (30)	1.76
7	1 (8)	0 (3)	1 (90)	0 (40)	0.49
8	0 (6)	1 (4)	-1 (30)	0 (40)	0.78
9	1 (8)	-1 (2)	0 (60)	0 (40)	0.57
10	1 (8)	0 (3)	0 (60)	1 (50)	0.48
11	0 (6)	-1 (2)	-1 (30)	0 (40)	2.03
12	0 (6)	1 (4)	1 (90)	0 (40)	1.68
13	0 (6)	0 (3)	1 (90)	1 (50)	1.25
14	-1 (4)	0 (3)	1 (90)	0 (40)	0.98
15	0 (6)	1 (4)	0 (60)	-1 (30)	1.28
16	1 (8)	0 (3)	0 (60)	-1 (30)	1.39
17	1 (8)	0 (3)	-1 (30)	0 (40)	1.09
18	-1 (4)	0 (3)	0 (60)	1 (50)	0.95
19	1 (8)	1 (4)	0 (60)	0 (40)	1.23
20	0 (6)	-1 (2)	1 (90)	0 (40)	0.89
21	-1 (4)	0 (3)	-1 (30)	0 (40)	0.66
22	0 (6)	-1 (2)	0 (60)	1 (50)	0.82
23	0 (6)	0 (3)	1 (90)	-1 (30)	1.29
24	-1 (4)	-1 (2)	0 (60)	0 (40)	1.26
25	0 (6)	0 (3)	0 (60)	0 (40)	3.96
26	0 (6)	0 (3)	-1 (30)	1 (50)	0.96
27	0 (6)	-1 (2)	0 (60)	-1 (30)	2.19

Table S3. Preferential enzymes and major active unit of enzyme formulation used

Enzyme	IUB No.	Major Units	Minimal Guaranteed Enzyme Activity[Units/g]
**Kemzyme ® Plus Concentrate dry,** (Kemin, Germany)	3.2.1.6	Endo-1,3 (4)-beta-glucanase (beta-glucanase) produced by *Aspergillus aculeatus* (CBS 589.94)	23 500
	3.2.1.4	Endo-1,4-beta-glucanase (cellulase) produced by *Trichoderma longibrachiatum* (CBS 592.94)	180 000
	3.2.1.1	Alpha-amylasa produced by *Bacillus amyloliquefaciens* (DSM 9553)	4 000
	3.4.24.28	Bacillolysine (protease) produced by *Bacillus amyloliquefaciens* (DSM 9554)	17 000
	3.2.1.8	Endo-1,4-beta-xylanase (xylanase) *Trichoderma viride* (NIBH FERM BP 4842)	350 000

1 U of Endo-1,3(4)-beta-glucanase is the amount of enzyme which liberates 0,0056 micromoles of reducing sugars (glucose equivalents) from barley beta-glucan per minute at pH 7,5 and 30°C.

1 U Endo-1,4-beta-glucanase is the amount of enzyme which liberates 0,0056 micromoles of reducing sugars (glucose equivalents) from carboxymethylcellulose per minute at pH 4,8 and 50°C

1 U of Alfa-amylase is the amount of enzyme which hydrolyses 1 micromole of glucosidic linkages from water insoluble cross-linked starch polymer per minute at pH 7,5 and 37°C

1 U of Bacillolysin is the amount of enzyme which makes 1 microgram of azo-casein soluble in trichoracetic acid per minute at pH 7,5 and 37°C.

1 U of Endo-1,4-beta-xylanase is the amount of enzyme which liberates 0,0067 micromoles of reducing sugars (xylose equivalents) from birchwood xylan per minute at pH 5,3 and 50°C.

1 FTU is the amount of enzyme which liberates 1 micromole of inorganic phosphate per minute from a sodium phytate substrate at pH 5,5 and 37 °C.

Table S4. MRM transitions, collision energy, Q1, Q3 and dwell time for investigated phenolic compounds.

Compounds	t_R [min]	MRM	Collision Energy [eV]	Q1 [eV]	Q3 [eV]	Dwell Time [msec]
**Phenolic acids**						
Gallic acid	2.138	169-124; 169-79	35	10	45	22
Salicylic acid	3.961	137-93; 137-65	35	10	45	26
p-Coumaric acid	3.450	163-119; 163-93	35	10	45	26
Chlorogenic acid	2.681	353-191; 353-85	35	10	45	22
Caffeic acid	3.036	179-135; 179-89	35	10	45	26
Syringic acid	3.118	179-135; 179-89	35	10	45	22
Ferulic acid	3.566	193-134; 193-89	35	10	45	26
Protocatechuic acid	2.482	153-108; 153-91	35	10	45	26
Sinapic acid	3.553	223-121; 223-149	35	10	45	26
4-Hydroxybenzoic acid	2.890	137-65; 137-108	35	10	45	22
**Flavonoids**						
Flavone	5.452	223-121; 223-77	-35	-10	-45	22
Fisetin	3.928	285-121; 285-135	35	10	45	26
Kaempferol	4.811	285-255; 285-239	35	10	45	26
Apigenin	4.730	269-117; 269-151	35	10	45	26
Luteolin	4.359	285-133; 285-107	35	10	45	26
Rutin	3.447	609-300; 609-271	35	10	45	26
Quercetin	4.382	301-227; 301-161	35	10	45	26
Naringin	3.528	579-271; 579-151	35	10	45	26
Naringenin	4.744	271-119; 271-107	35	10	45	26
Esculin	2.492	339-177; 339-133	35	10	45	26
Esculetin	3.053	177-89; 177-105	35	10	45	22
Biochanin A	5.666	283-211; 283-239; 283-132	35	10	45	26
Catechin	3.012	289-123; 289-109	35	10	45	22

Fig. S1. The single MRM chromatograms and full chromatogram for investigated phenolic compounds.

Flavone

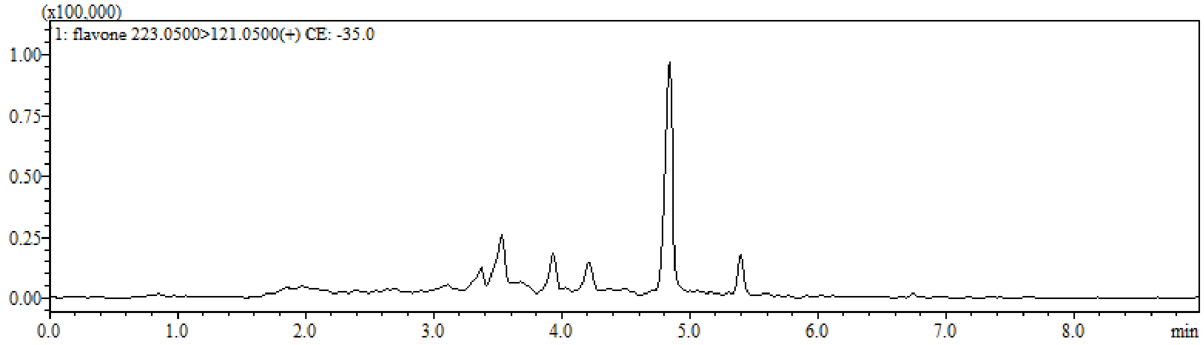

Fisetin

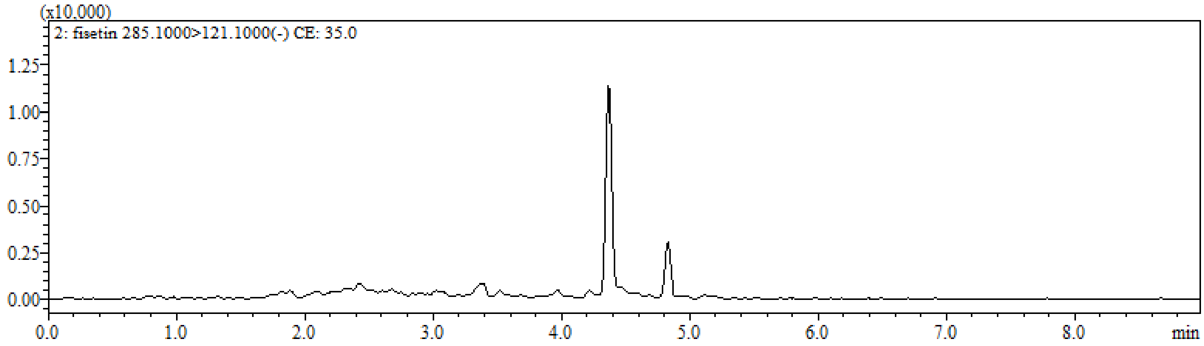

Chlorogenic acid

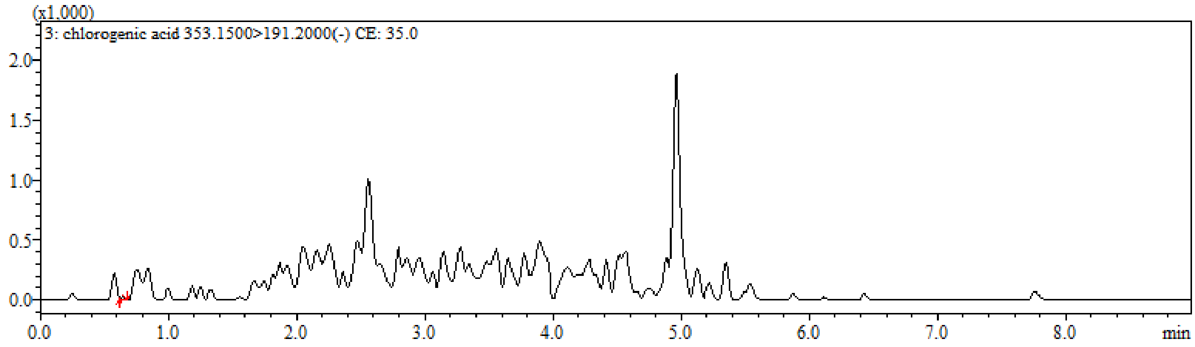

p-Coumaric acid

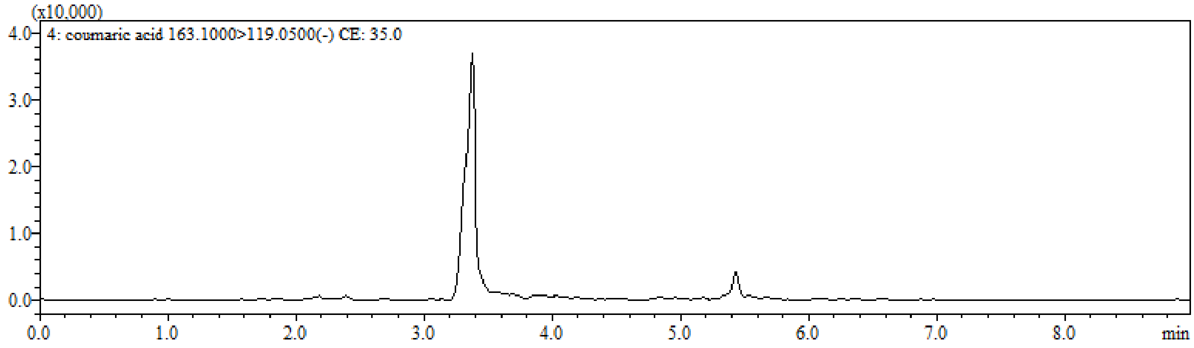

Salicylic acid

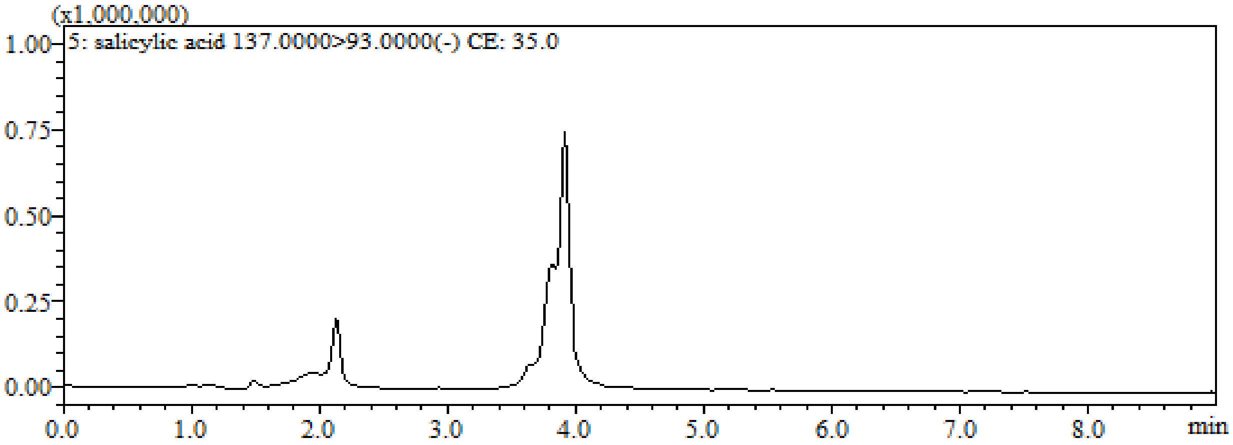

Gallic acid

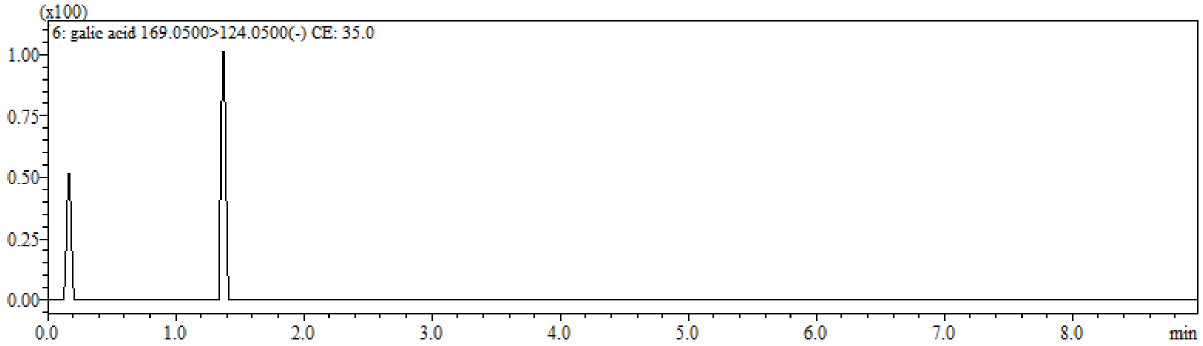

Caffeic acid

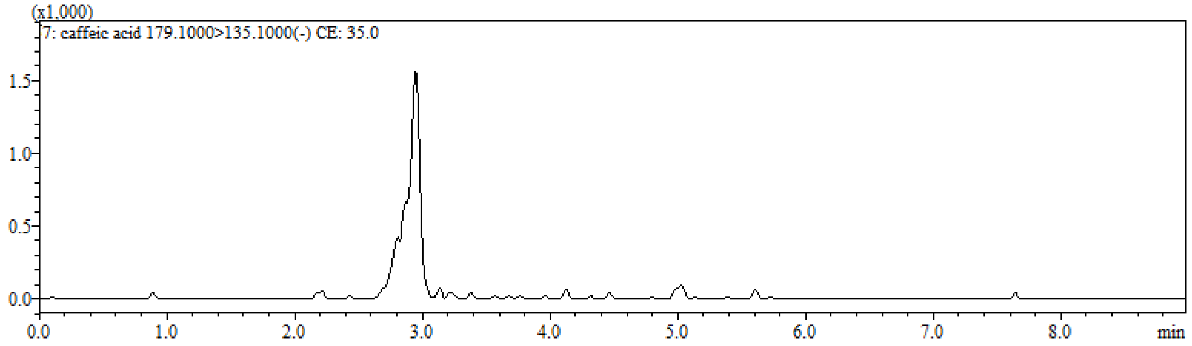

Syringic acid

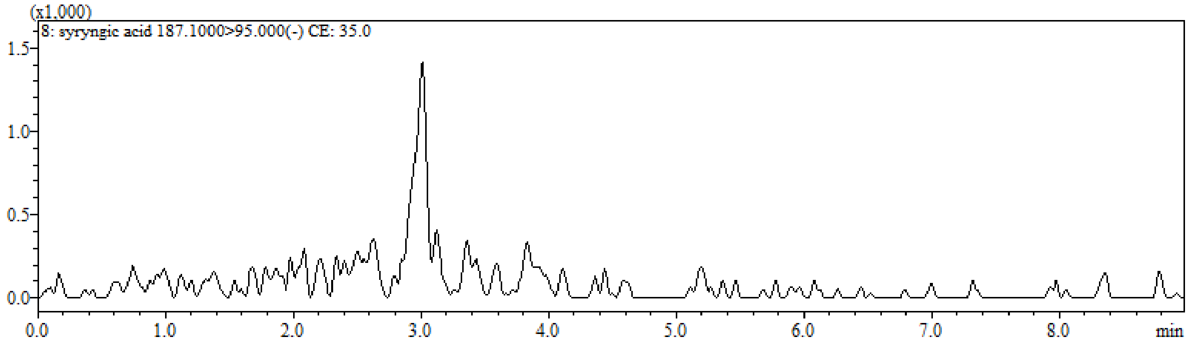

Ferulic acid

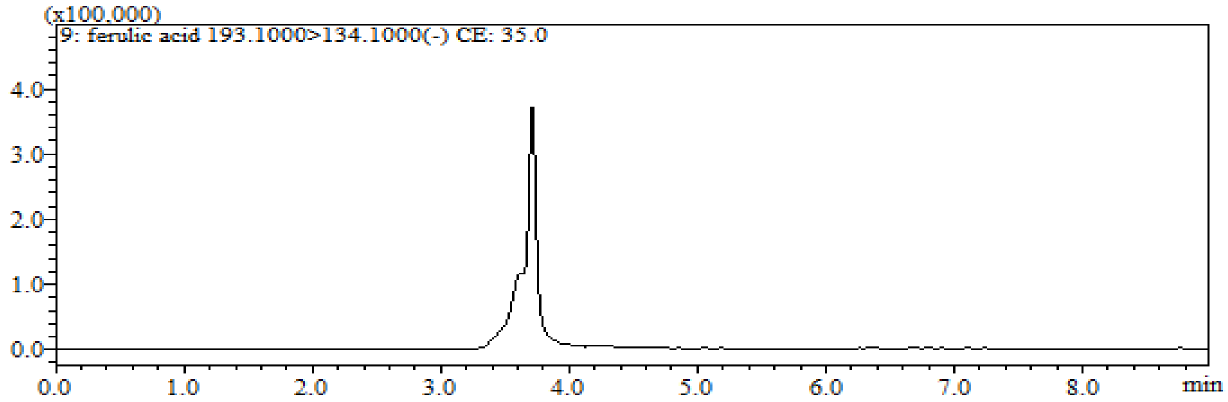

Protocatechuic acid

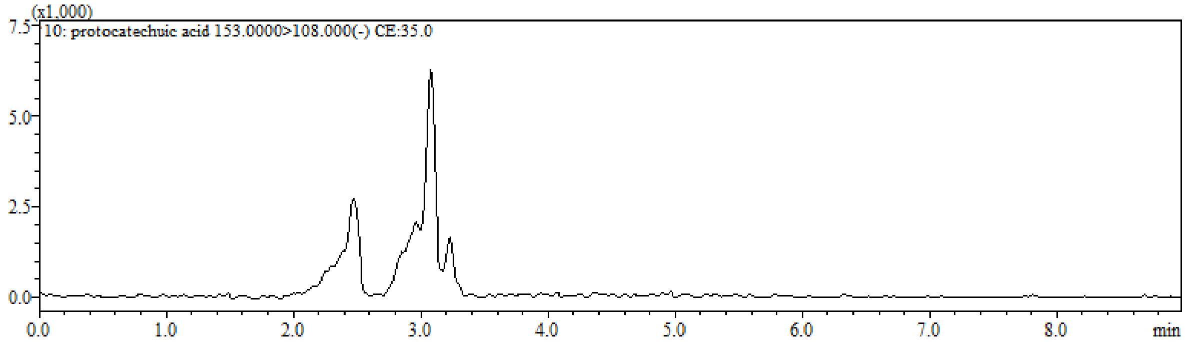

Sinapic acid

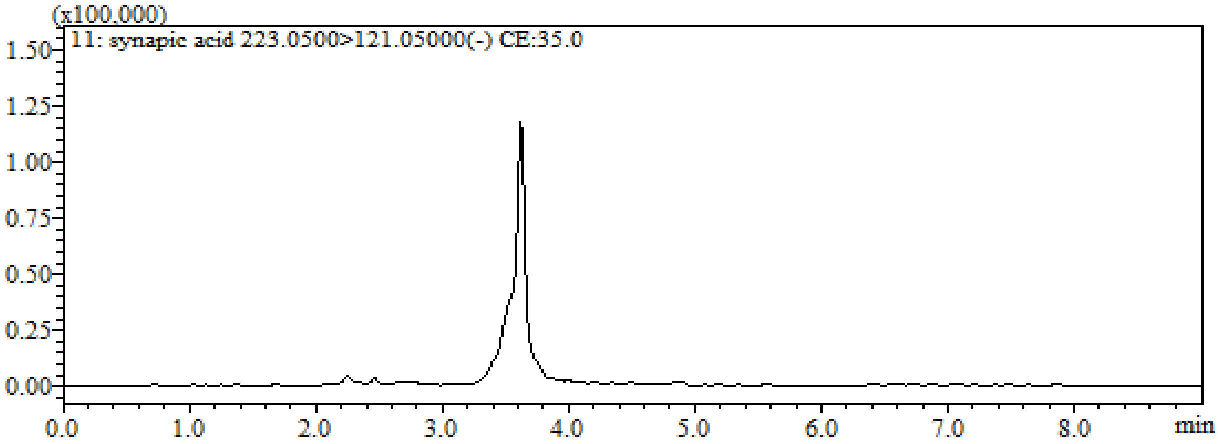

4-Hydroxybenzoic acid

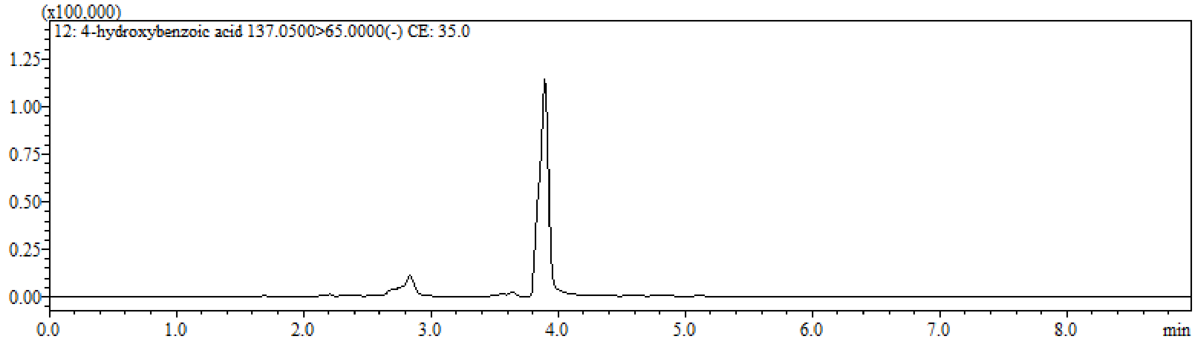

Catechin

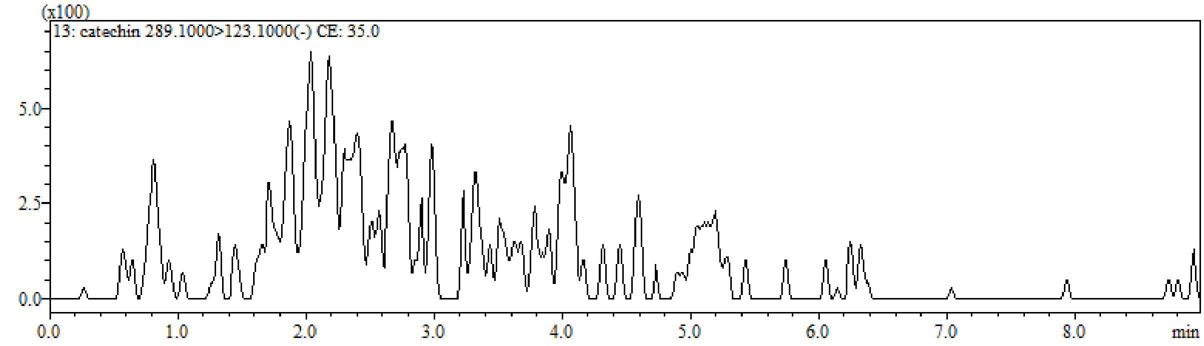

Biochanin a

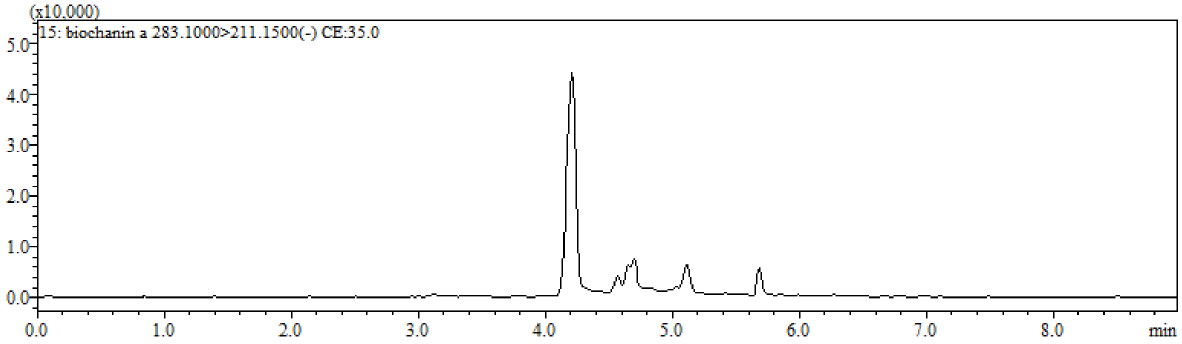

Esculetin

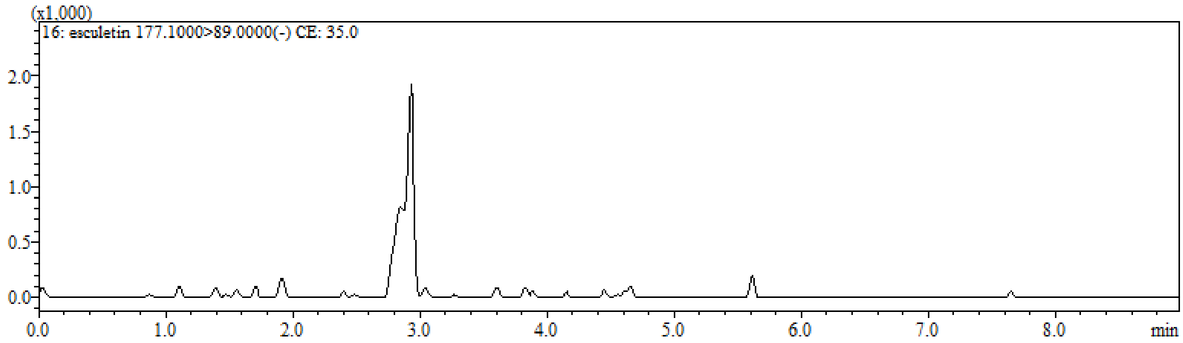

Esculin

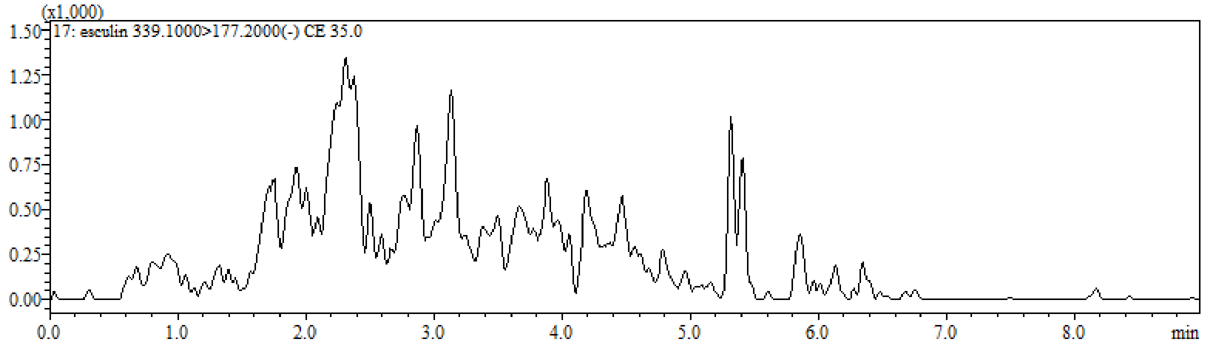

Naringenin

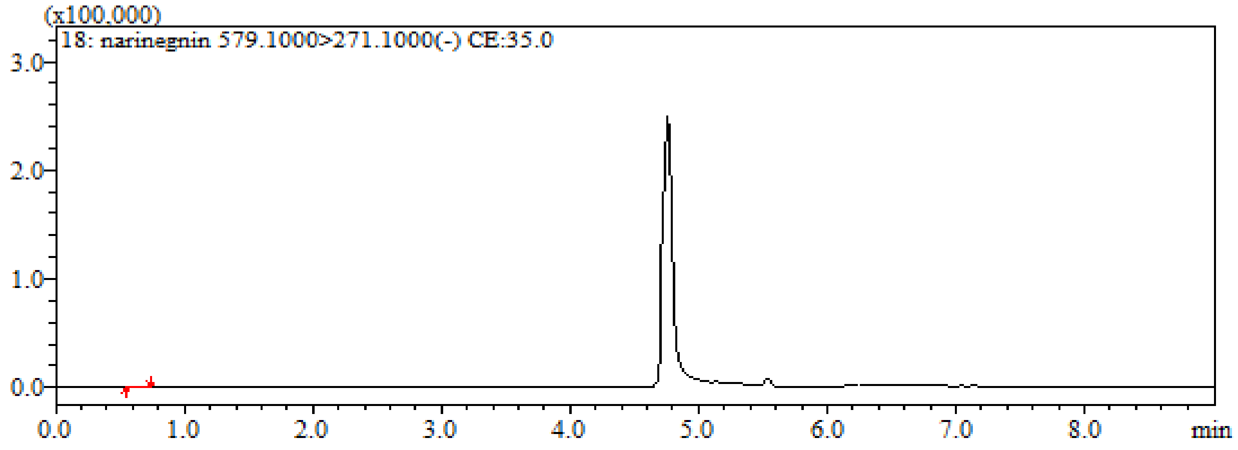

Naringin

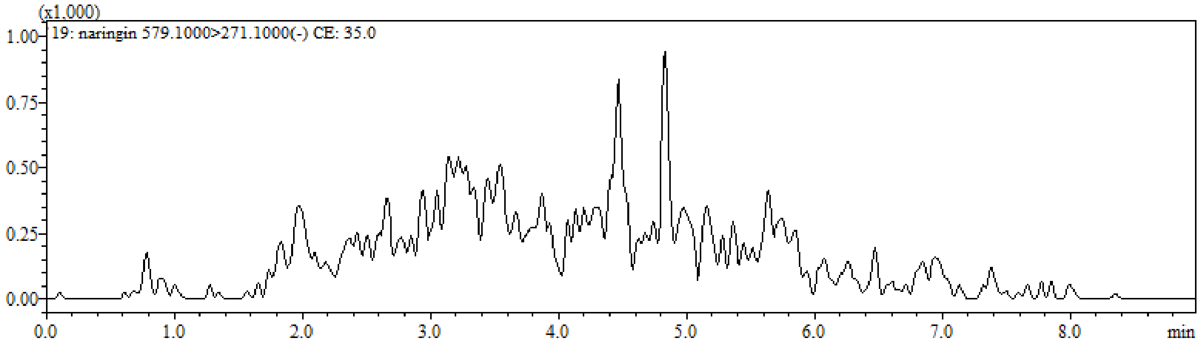

Quercetin

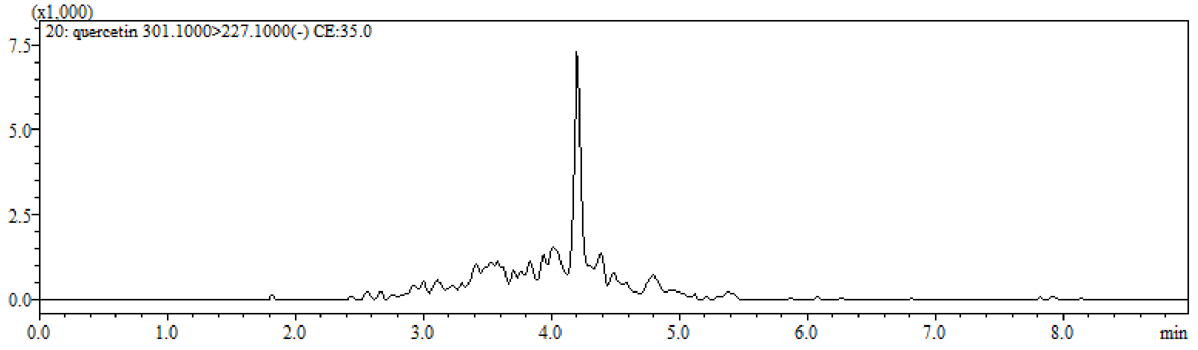

Rutin

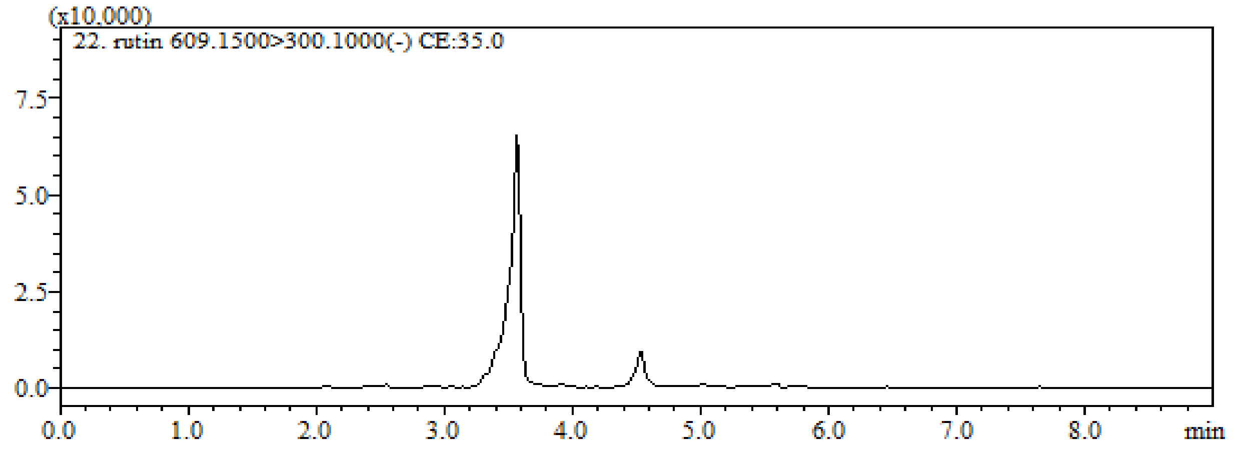

Luteolin

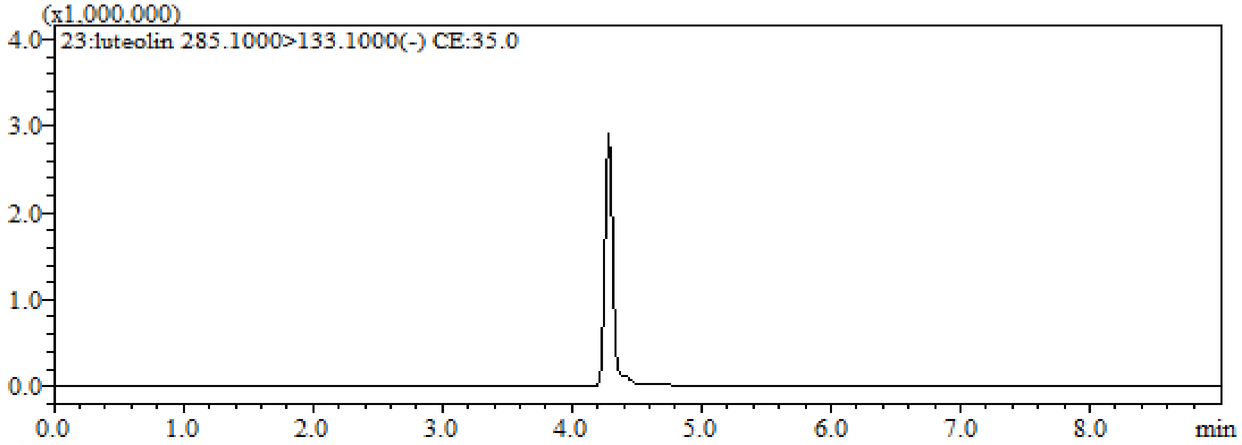

Apigenin

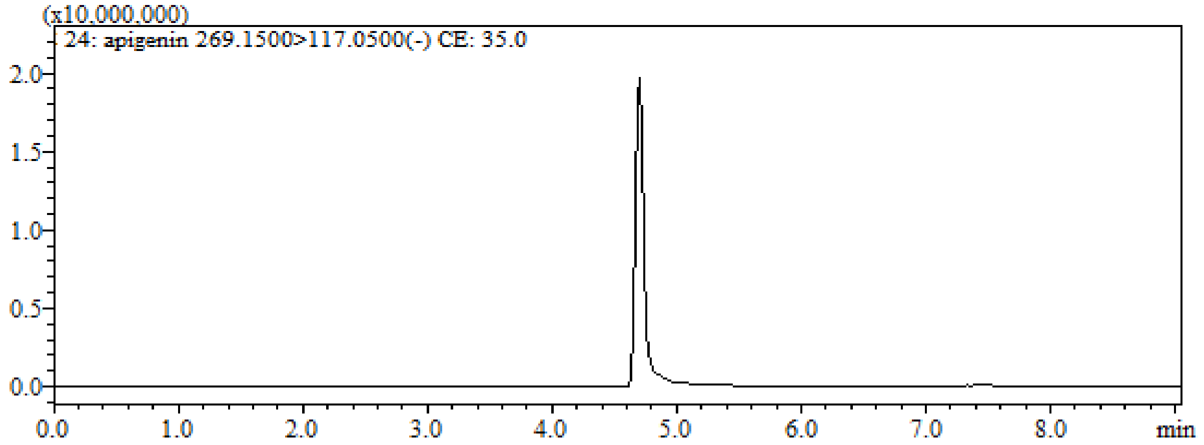

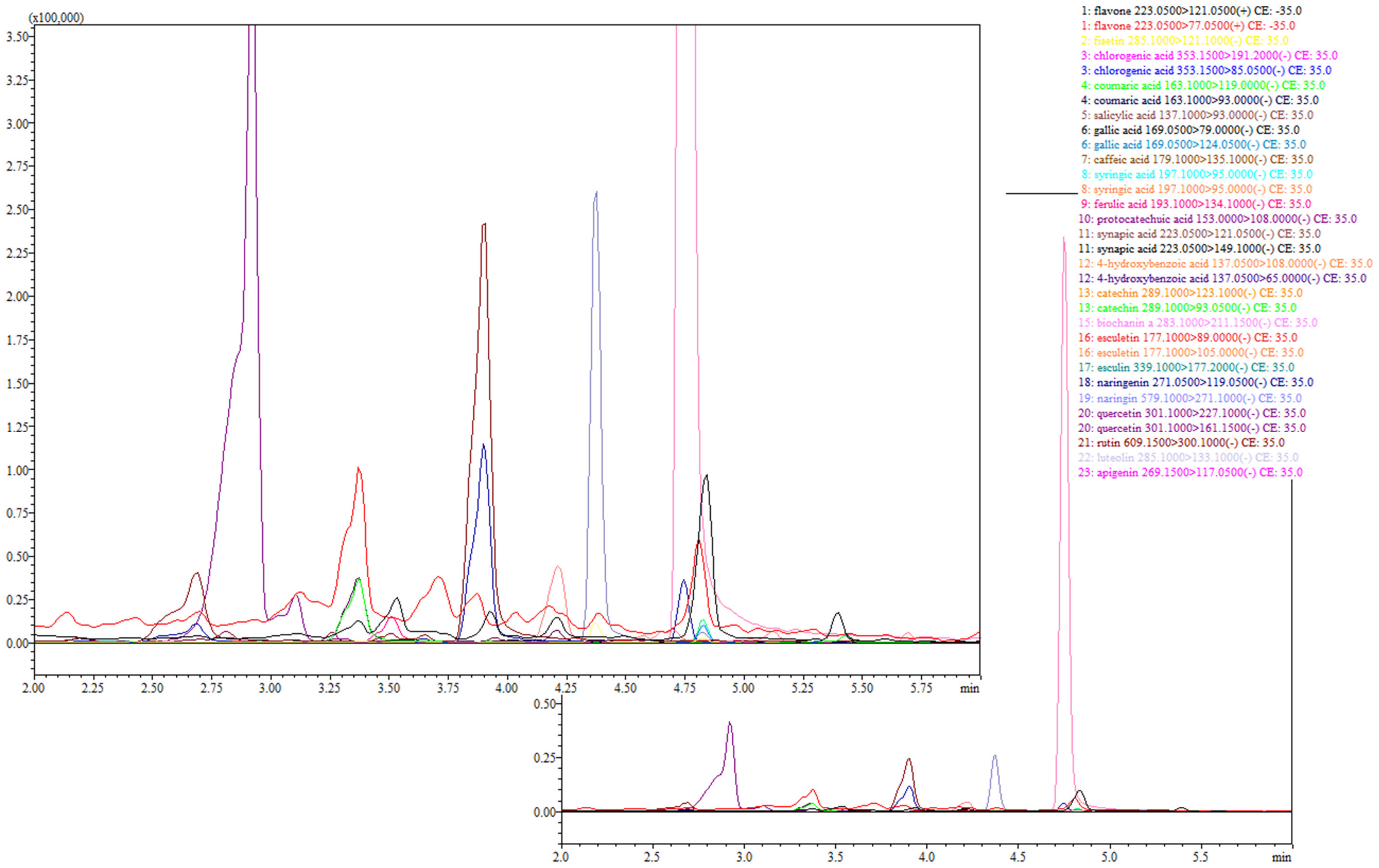
(x100,000)
min
1: flavone 223.0500>121.0500(+) CE: -35.0
1: flavone 223.0500>77.0500(+) CE: -35.0
2: fisetin 285.1000>121.1000(-) CE: 35.0
3: chlorogenic acid 353.1500>191.2000(-) CE: 35.0
3: chlorogenic acid 353.1500>85.0500(-) CE: 35.0
4: coumaric acid 163.1000>119.0000(-) CE: 35.0
4: coumaric acid 163.1000>93.0000(-) CE: 35.0
5: salicylic acid 137.1000>93.0000(-) CE: 35.0
6: gallic acid 169.0500>79.0000(-) CE: 35.0
6: gallic acid 169.0500>124.0500(-) CE: 35.0
7: caffeic acid 179.1000>135.1000(-) CE: 35.0
8: syringic acid 197.1000>95.0000(-) CE: 35.0
8: syringic acid 197.1000>95.0000(-) CE: 35.0
9: ferulic acid 193.1000>134.1000(-) CE: 35.0
10: protocatechuic acid 153.0000>108.0000(-) CE: 35.0
11: synapic acid 223.0500>121.0500(-) CE: 35.0
11: synapic acid 223.0500>149.1000(-) CE: 35.0
12: 4-hydroxybenzoic acid 137.0500>108.0000(-) CE: 35.0
12: 4-hydroxybenzoic acid 137.0500>65.0000(-) CE: 35.0
13: catechin 289.1000>123.1000(-) CE: 35.0
13: catechin 289.1000>93.0500(-) CE: 35.0
15: biochanin a 283.1000>211.1500(-) CE: 35.0
16: esculetin 177.1000>89.0000(-) CE: 35.0
16: esculetin 177.1000>105.0000(-) CE: 35.0
17: esculin 339.1000>177.2000(-) CE: 35.0
18: naringenin 271.0500>119.0500(-) CE: 35.0
19: naringin 579.1000>271.1000(-) CE: 35.0
20: quercetin 301.1000>227.1000(-) CE: 35.0
20: quercetin 301.1000>161.1500(-) CE: 35.0
21: rutin 609.1500>300.1000(-) CE: 35.0
22: luteolin 285.1000>133.1000(-) CE: 35.0
23: apigenin 269.1500>117.0500(-) CE: 35.0
